# How task difficulty and academic self-efficacy impact retrieval practice guidance

**DOI:** 10.3389/fpsyg.2023.1260084

**Published:** 2023-11-22

**Authors:** Chenchen Liao, Jinkun Zhang

**Affiliations:** ^1^Students' Mental Health Center, Minnan University of Science and Technology, Quanzhou, China; ^2^School of Psychology, Fujian Normal University, Fuzhou, China

**Keywords:** retrieval practice, guidance, self-regulated learning, task difficulty, academic self-efficacy

## Abstract

Retrieval practice can enhance learning but is rarely used in self-regulated learning. Although explicit retrieval practice guidance (RPG)—which helps students use retrieval correctly—can improve learning outcomes, however, task difficulty and differences in academic self-efficacy (ASE) may influence retrieval practice decisions and learning performance, which were not considered in previous researches. The purpose of this study was to explore whether RPG produces different effects due to task difficulty and ASE. In Experiment 1, participants studied tasks with varying difficulty levels, some of which were guided. Results showed that RPG could enhance learning through increased retrieval practice, and participants engaged in more retrieval for difficult tasks. In Experiment 2, participants with different degrees of ASE learned tasks under guidance. Participants with high ASE persisted better on different tasks. Hence, task difficulty can affect retrieval practice decisions, and ASE increases persistence in retrieval practice. The implications of the findings for students’ use of RPG are discussed in this article.

## 1. Introduction

The effectiveness of retrieval-based learning has been confirmed by numerous studies (Adesope et al., 2017; McDermott, 2021). When learning has reached a certain level, retrieval practice can promote learning better than more elaborate strategies such as concept-mapping or learning-by-teaching (Karpicke and Bauernschmidt, 2011; Koh et al., 2018), and long-term retention is aided by frequent retrieval during learning (Rawson and Dunlosky, 2011; Vaughn and Rawson, 2011).

However, learners use self-tests primarily to assess their mastery of knowledge rather than to improve their memory (Kornell and Bjork, 2007). People may experience frustration due to the effort of retrieval (Bjork et al., 2013), and are unlikely to discover the direct effectiveness of retrieval practice through their own experiences (Tullis et al., 2013), which often cause learners to avoid retrieval practice and to prefer re-reading on memory tasks (Carpenter et al., 2020; Tullis and Maddox, 2020). Given a lack of metacognitive knowledge, explicit learning strategy guidance may be warranted (Dignath and Veenman, 2021). While interventions have all yielded mixed results. Ariel and Karpicke (2018) provided college students with instructions for retrieval practice, informing them of the advantages of retrieval strategies and how to use them correctly to increase their frequency of use in the learning process. The results showed that students under guidance practiced retrieval significantly more than the control group and were less likely to stop learning after their correct retrieval, they also outperformed the control group on a cued-recall test. The hopeful results encouraged some researchers that are interested in finding out whether these effects could be generalized in an authentic education environment. However, the intervention had no positive effects on self-regulated use of retrieval practice (except for transfer session) and test performance (Broeren et al., 2021), which was the same with another intervention occur in the context of courses (Rodriguez et al., 2021). While some interventions showed that participants reported they had enhanced knowledge of the effective learning strategies and increased the use of practice testing (Biwer et al., 2020; McCabe et al., 2021), but there was still a gap between knowledge and actual use (Biwer et al., 2020). Therefore, some researchers separated the intention to use retrieval practice from its actual use, provided students procedural metacognitive knowledge (that retrieval practice can be implemented flexibly in various formats) and reduced participants’ perceived cost, compared to only declarative metacognitive knowledge, there were also no effects of their intervention on reported use of retrieval practice in the long term (Wang et al., 2023). Broeren et al. (2023) provided students either strategy instructions on retrieval practice (RP condition), strategy instructions and metacognitive email support (RP++) or no support (control), results showed a small but significant effect on retrieval practice use for the RP++ condition as compared to control, no significant differences were found between RP++ and RP.

Until now there is limited evidence to show what affects students’ retrieval practice decisions under guidance. We have to acknowledge that the effect of interventions could be influenced by environment(online, classroom or lab-experiment), we also notice that the materials in these interventions are different in difficulty, like word-pair, key concept etc. However, previous researches did not consider the aspect of task difficulty; it remains unknown whether retrieval practice decisions would be influenced by task difficulty. Since easy concepts/ideas often require fewer successful retrieval practice trials than difficult concepts/ideas to attain similar retention advantages, researchers assumed that students would engage in more retrieval practice for subjectively harder materials than for subjectively easier materials (Vaughn et al., 2013). A few studies presented participants with items of varying levels of difficulty and found that participants were inclined to choose testing more often for easier items and tended to re-study difficult items more often (Karpicke et al., 2009; Toppino et al., 2018; Tullis et al., 2018). This may be because learners feel more confident in retrieving easy items and avoid retrieving difficult items that could lead to failure. Hence, retrieval practice guidance (RPG) and the level of task difficulty may simultaneously impact the learning effect. Based on the positive influence of retrieval practice strategies on learning, it is necessary to carry out research to understand and increase students’ use of retrieval practice strategies on tasks with different levels of difficulty. In view of this, Experiment 1 addressed the impact of task difficulty on the effect of RPG. Additionally, one potential limitation of previous interventions is that learning strategy use was measured by self-report only, which have been questioned that could not know the actual use of participants when they are studying, due to potential demand characteristics. And one of them was lack the baseline group without intervention. It seems that a learning-strategies-only intervention (only declarative metacognitive knowledge) about retrieval practice make no differences with conditions (i.e., additional procedural metacognitive knowledge, RP++, a learning-strategies-plus-behavior-change intervention) that aimed to behavior change of participants, which may because the conditions caused high cognitive load. In view of this, we utilized behavioral measures and set a no-intervention control group, experiment 1 addressed the impact of task difficulty on the effect of RPG, the guidance was based on the instructions from Ariel and Karpicke’s experiment.

Moreover, during the self-regulated learning (SRL) process, metacognitive strategies interact with motivational and cognitive processes (Boekaerts, 1999). After providing learners with repeated retrieval guidance at the metacognitive level, whether they decide to adopt the cognitive strategy of repeated retrieval depends on their motivation to a certain extent. Dunlosky and Rawson (2015) asserted that participants may become less engaged over time, motivation for repeated retrieval may decline, and students with different beliefs and orientations may act differently in terms of persistence. By affecting the initiation and persistence of learning activities, motivational processes including self-efficacy and goal setting contribute to SRL (Efklides, 2011).

According to social cognitive theory, people’s decision-making, perseverance, and attitudes toward challenges are all influenced by their level of self-efficacy. Students with high academic self-efficacy (ASE) have greater task persistence than students with low ASE (Pajares, 2002). As a specific application of self-efficacy in the learning field, ASE can significantly predict learning engagement (Lin and Xuji, 2020). So far, no research directly exploring whether and how retrieval practice decisions can be influenced by learners’ academic self-efficacy. Recent studies have examined the connection between retrieval practice and self-efficacy. The findings show that, in comparison to neutral feedback, negative feedback diminishes self-efficacy, which in turn results in worse memory performance. Reduced self-efficacy results in less perseverance (e.g., decreased memory search), which causes poor learning outcomes (Frankenstein et al., 2022). Wang et al. (2023) measured self-efficacy ratings of using retrieval practice in their own studying after interventions, and found that reported intention to use was positively predicted by students’ self-efficacy for engaging in retrieval practice in the short term, but the intervention did not increase the self-efficacy or reduce perceived cost in the long term. One issue is that the study did not conduct a pretest of participants’ self-efficacy, so we cannot directly compare changes within the group, nor understand the stable self-efficacy (e.g., ASE) of participants themselves. There were participants with different levels of self-efficacy, that short term metacognitive interventions may not be sufficient to change in the long term. Thus, it is pertinent to explore whether ASE would affect decision-making about retrieval during SRL. Retrieval practice is considered a desirable kind of difficulty (Bjork et al., 2013) as it informs learners while they retrieve information at least three times; this is different from the usual “one-and-done” method whereby students cease studying content after only briefly recalling it once. Retrieval practice requires higher motivation, confidence, and energy. In addition, the challenge of retrieval varies for tasks with different degrees of difficulty. For word pairs with higher associated strength, the availability of the target word is stronger; for word pairs with lower association, more effort is required, and retrieval failure is more likely to occur. In the context of providing guidance, it is unclear whether learners with different levels of ASE would act differently when performing tasks with different levels of difficulty. The answer to this question relates to the prediction that students are more likely to use a self-testing strategy when RPG is applied to the classroom. Based on this, in Experiment 2, we further investigated whether guidance applies to individuals with different levels of ASE.

We aimed to explore whether RPG can significantly increase learners’ use of self-testing and whether the difficulty of the task would influence the guidance effect, as well as to better understand whether learners with different levels of ASE would display differences in learning under the guidance provided. The findings provide evidence for educators about how to guide students to use retrieval practice more effectively while taking into account the role of individual differences in ASE and laying a foundation for developing unique interventions for different students.

## 2. Experiment 1

### 2.1. Method

#### 2.1.1. Participants

The sample comprised 60 undergraduates with a mean age of 20.82 years (SD = 1.41, range = 18–24 years). After completing the experiment, the participants received a small gift as a reward.

#### 2.1.2. Design

The experiment involved a 2 × 3 mixed design with RPG (guidance vs. no guidance) and task difficulty (easy, medium, difficult). We manipulated the guidance between participants; half studied under guidance, whereas the other half did not. Additionally, we manipulated task difficulty among the participants, all of whom learned tasks with different degrees of difficulty. The participants were randomly assigned to the experimental groups.

#### 2.1.3. Materials

A total of 45-word pairs were used. The specific selection process was as follows: We chose 100 pairs of neutral words (word frequency: 0.89–0.30%) from the Modern Chinese Frequency Dictionary and randomly selected 74 college students (not participating in the formal experiment) to rate the materials. The participants evaluated the association of each word pair using a 7-point scale. We identified three groups of related words and performed a one-way analysis of variance (ANOVA) for the association of word pairs. The associations between the three groups were significantly different [*F*(2,72) = 482.529, *p* < 0.000].

#### 2.1.4. Procedure

The experimental materials were randomly presented on a computer, which the participants were told to operate according to the instructions. The group under RPG received guidance translated from the research used in Ariel and Karpicke (2018). RPG informs students through picture and text that the mnemonic benefits of retrieval practice over restudy, and how to use retrieval practice in self-regulated learning to maximize their performance, that is, each word pair should be correctly recalled three times. The control group did not receive guidance; the participants in this group were only asked to remember the words they had learned as much as possible during the final recall test. At the beginning of the experiment, a white gaze signal, +, was displayed in the center of the screen for 1 s; then, 45 pairs of related words were presented at a rate of 8 s per word. After the word pair disappeared, the participants could choose to take a retrieval, reread, or drop items that they would not practice any more by pressing a button. The learning phase was entered until all word pairs were presented. If the participants chose to re-study an item, the word pair would be displayed for 8 s. If they chose to retrieve, they would only be shown clue words; participants were required to write the target words (6 s) on paper and to review the feedback for 2 s. They would not learn the word pair in the learning phase if they chose to abandon an item. All word pairs were presented in a random order for each trial. In this phase, participants could also choose the method of learning word pairs in the next trial until all word pairs were dropped and the learning phase ended. Participants then needed to watch a 15-min video before taking the final recall test; the video was irrelevant to the memory task and was designed to serve as a distraction. In the final test, the cue words were displayed and participants were asked to recall the target words within an unlimited time frame.

### 2.2. Results

#### 2.2.1. Number of retrieval attempts during learning

Table 1 portrays the number of retrieval practice attempts for each group of items during the learning phase. A repeated measures ANOVA with the factors of RPG (guidance vs. no guidance) and task difficulty (easy, medium, difficult) revealed significant main effects of RPG, *F*(1,57) = 37.06, *p* < 0.05, η_p_^2^ = 0.39. Participants under guidance chose to test themselves more than participants who received no guidance. We also found a main effect of task difficulty, *F*(1,57) = 7.23, *p* < 0.05, η_p_^2^ = 0.11; the number of retrievals under the medium and difficult conditions were significantly greater than the number of retrievals for simple tasks, while the differences in retrievals for medium versus difficult conditions were not significantly different. No interactions were reliable [*F*(1,57) = 0.11, *p* > 0.05, η_p_^2^ = 0.002].

**Table 1:** The number of retrieval practice attempts as a function of retrieval practice guidance and task difficulty.

	Task difficulty		
	Easy	Medium	Difficult
Guidance	2.20 (0.92)	2.39 (0.89)	2.41 (0.81)
No guidance	1.06 (0.66)	1.28 (0.64)	1.25 (0.69)

The standard error of the mean is in parentheses.

#### 2.2.2. Final recall rates

Table 2 depicts the final correct recall rates under different states of RPG (guidance vs. no guidance) and levels of task difficulty. We conducted a repeated measures ANOVA on the final recall data with the factors of RPG (guidance vs. no guidance) and task difficulty (easy, medium, difficult). RPG had a significant main effect on final recall, *F*(1,57) = 13.32, *p* < 0.05, η_p_^2^ = 0.19; the group under guidance recalled significantly more target words than the control group. We also found a main effect of task difficulty, *F*(1,57) = 15.42, *p* < 0.05, η_p_^2^ = 0.21; the correct recall rate of the easy task was significantly higher than that of the medium and difficult tasks, and the correct recall rate of the medium task was significantly higher than that of the difficult task. We detected no interaction [*F*(1,57) = 1.98, *p* > 0.05, η_p_^2^ = 0.03].

**Table 2:** The final correct recall rates as a function of retrieval practice guidance and task difficulty.

	Task difficulty		
	Easy	Medium	Difficult
Guidance	0.95 (0.09)	0.92 (0.10)	0.88 (0.15)
No guidance	0.83 (0.18)	0.79 (0.21)	0.70 (0.26)

The standard error of the mean is in parentheses.

Consistent with the results found in the original study by Ariel and Karpicke (2018). In brief, the results of Experiment 1 indicated that retrieval practice instructions effectively increased participants’ use of retrieval and boosted their learning performance, which is in line with the intention of the tactics. Simultaneously, task difficulty affected the learners’ retrieval decisions. Specifically, they tended to retrieve more during the medium and difficult tasks, but their learning effect was worse than during the easy task.

To further predict the use of retrieval practice by learners with different levels of ASE under guidance conditions, in Experiment 2, we investigated the learning situation of individuals with different levels of ASE on tasks with different degrees of difficulty.

## 3. Experiment 2

### 3.1. Method

#### 3.1.1. Participants

Sixty undergraduates participated in Experiment 2; their mean age was 21.15 years (SD = 1.44, range = 18–24 years), and none of them had participated in Experiment 1. After completing the experiment, they received a small gift as a reward.

#### 3.1.2. Design

Experiment 2 involved a 2 × 3 mixed design, with the factors of ASE (high vs. low) and task difficulty (easy, medium, difficult). We manipulated ASE between the participants and task difficulty within the participants.

#### 3.1.3. Materials

The 45-word pairs were the same as in Experiment 1. We introduced one difference in the materials and used an ASE questionnaire (Yusong, 2000). The questionnaire covered the relevant dimensions of the ASE questionnaire developed by Pintrich and De Groot (1990), which includes two dimensions: self-efficacy of learning ability (competence efficacy) and self-efficacy of learning behavior (behavioral efficacy). The questionnaire was validated for university students; the Cronbach’s α of the competence efficacy and behavioral efficacy subscales were 0.820 and 0.752, respectively. The questionnaire had a total of 22 items rated on a 7-point scale, with no reverse scoring: the higher the score was, the greater was the learner’s sense of ASE.

#### 3.1.4. Procedure

Before the experiment, we randomly selected 200 college students to respond to the ASE questionnaire, which was administered online. After excluding invalid responses, we obtained a total of 185 valid responses. We added the scores of each item to obtain the total scores, which were then sorted in descending order. The top (bottom) 27% in terms of total scores were classified in the high (low) ASE group. The other procedures were the same as those used in Experiment 1.

### 3.2. Results

#### 3.2.1. Number of retrieval attempts during learning

Table 3 outlines the number of retrieval attempts among participants with different levels of ASE for the tasks performed during the learning phase. We conducted a repeated measures ANOVA with the factors of ASE (high vs. low) and task difficulty (easy, medium, difficult). ASE had a significant main effect on the number of retrieval attempts [*F*(1,57) = 8.70, *p* < 0.05, η_p_^2^ = 0.13] and task difficulty [*F*(1,57) = 20.60, *p* < 0.05, η_p_^2^ = 0.26]. Participants with high ASE chose to self-test more than those with low ASE did, and the number of retrievals during the medium and difficult tasks was significantly greater than the number of retrievals for the easy task, while there were no significant differences between retrievals for medium and difficult tasks. ASE had no interaction with task difficulty [*F*(1,57) = 0.08, *p* > 0.05, η_p_^2^ = 0.001].

**Table 3:** The number of retrieval practice attempts among participants with different levels of academic self-efficacy on tasks with different levels of difficulty.

Academic self-efficacy	Task difficulty		
	Easy	Medium	Difficult
High	1.72 (0.84)	2.03 (0.75)	2.16 (0.83)
Low	1.16 (0.85)	1.45 (0.86)	1.54 (0.81)

The standard error of the mean is in parentheses.

#### 3.2.2. Final recall rates

Table 4 displays the recall performance rates on the final test for participants with different levels of ASE. A repeated measures ANOVA on the final recall data revealed a significant main effect of ASE, *F*(1,57) = 5.87, *p* < 0.05, η_p_^2^ = 0.09; the correct recall rate of participants with high ASE was significantly higher than that of participants with low ASE. The main effect of task difficulty was significant, *F*(1,57) = 4.75, *p* < 0.05, η_p_^2^ = 0.08, and the correct recall rate of the easy task was significantly higher than that of the medium and difficult tasks, whereas there were no significant differences in the correct recall rates between medium and difficult tasks. We did not observe any interaction, *F*(1,57) = 0.53, *p* > 0.05, η_p_^2^ = 0.009.

**Table 4:** The final correct recall rates of participants with different levels of academic self-efficacy on tasks with different levels of difficulty.

Academic self-efficacy	Task difficulty		
	Easy	Medium	Difficult
High	0.90 (0.12)	0.87 (0.21)	0.84 (0.20)
Low	0.79 (0.19)	0.77 (0.24)	0.70 (0.29)

The standard error of the mean is in parentheses.

The results of Experiment 2 suggest that ASE has an impact on the effect of RPG. Compared to learners with low ASE, learners with high ASE tended to retrieve more items with varying levels of difficulty and recalled a greater proportion of items which is partially consistent with the viewpoint of Wang et al. (2023), that is to say, learners with high ASE not only had higher intension of retrieval practice, but also more actual use.

## 4. Discussion

We examined the effect of RPG on learning as well as the role of different levels of task difficulty and ASE. Three major findings emerged from the two experiments. First, RPG can significantly enhance students’ learning. Second, given sufficient time, learners tended to perform more retrievals on the difficult tasks than on easy ones. Third, under RPG, learners with high ASE engaged in more retrieval practice and had better recall performance. These outcomes signal that the RPG intervention may be more effective for learners with high ASE; however, at the same time, the impact of task difficulty on retrieval decisions should be considered. These results are consistent with the finding that retrieval practice intervention can improve SRL (Ariel and Karpicke, 2018). Indeed, compared to learners who did not receive additional guidance, the learners who received guidance were more likely to continue studying after successful retrieval. This was mainly because RPG might correct the learner’s understanding that “retrieval is a self-assessment tool” at the metacognitive level, and help them to realize that repeated retrieval can enhance memory retention.

Table 5 displays the number of retrieval practice attempts and final correct recall rates of all participants. Both experiments indicated that learners preferred to test themselves more on difficult tasks than on easy ones, which is inconsistent with previous research. The discrepancy-reduction model may explain this outcome (Thiede and Dunlosky, 1999; Son and Metcalfe, 2000). According to the model, when learners have sufficient time and opportunities for self-pacing, they will allocate more time to studying difficult tasks to optimize test performance. If we infer from the results of Experiment 1, from the perspective of materials, previous research used key concepts, which require more understanding rather than memorization, as they can be described using other vocabulary and are relatively simple in terms of difficulty, students rarely engage in precise repeated retrieval in their daily learning (Broeren et al., 2021, 2023).Furthermore, providing immediate feedback increases the likelihood that learners will choose to test themselves instead of repeatedly reading (Rivers, 2021). This is because feedback enables learners to self-assess their level of mastery. On the one hand, feedback affirms the learner’s correct recall; on the other hand, if the learner experiences mistaken recall, there is still a chance to recode. Hence, repeated retrieval with feedback can increase intrinsic motivation to continue studying, potentially by providing a person with the impression of learning progress and the experience of competence (Abel and Bäuml, 2020), can increase students’ decisions to use it (Carpenter, 2023). And it also points to the opportunity for more automated/reactive testing techniques to be used in instruction, online instruction, in particular. Furthermore, providing immediate feedback increases the likelihood that learners will choose to test themselves instead of repeatedly reading (Rivers, 2021). This is because feedback enables learners to self-assess their level of mastery. On the one hand, feedback affirms the learner’s correct recall; on the other hand, if the learner experiences mistaken recall, there is still a chance to recode. Hence, repeated retrieval with feedback can increase intrinsic motivation to continue studying, potentially by providing a person with the impression of learning progress and the experience of competence (Abel and Bäuml, 2020).

**Table 5:** The number of retrieval practice attempts and final correct recall rates of all participants.

	Task difficulty			
		Easy	Medium	Difficult
Guidance	Attempts	2.20 (0.92)	2.39 (0.89)	2.41 (0.81)
	Recall rates	0.95 (0.09)	0.92 (0.10)	0.88 (0.15)
No guidance	Attempts	1.06 (0.66)	1.28 (0.64)	1.25 (0.69)
	Recall rates	0.83 (0.18)	0.79 (0.21)	0.70 (0.26)
High ASE	Attempts	1.72 (0.84)	2.03 (0.75)	2.16 (0.83)
	Recall rates	0.90 (0.12)	0.87 (0.21)	0.84 (0.20)
Low ASE	Attempts	1.16 (0.85)	1.45 (0.86)	1.54 (0.81)
	Recall rates	0.79 (0.19)	0.77 (0.24)	0.70 (0.29)

The standard error of the mean is in parentheses.

Dating back to previous SRL research on retrieval practice, although learners chose their own learning strategies, there were restrictions on the learning time, such as only one learning opportunity, which made it difficult for learners to estimate the direct benefits of retrieval practice and the risk of retrieval failure, especially in the case of difficult tasks. Repeated reading can increase learners’ confidence in maintaining memory, making them more inclined to re-read (Toppino et al., 2018; Tullis et al., 2018). We did not design the present study to investigate the impact of learning time and feedback on decisions regarding retrieval practice. Future research should continue to explore whether differences in learning time and feedback in SRL will lead to different retrieval practice choices. Additionally, although the learners performed more retrieval on the difficult and medium tasks, their final correct recall rates were significantly lower than that in the case of the easy task. This finding is in line with other studies (Vaughn et al., 2013; Lima et al., 2020) and refers to the item difficulty effect, which means that in a multi-sequence learning paradigm, after individuals learn related word pairs, they recall the target words with the hint of clue words; when the difficulty of items is distinguished, learners will find that compared to difficult items, there is a greater RPG for easy items. In the present study, even if the learning time was increased for difficult items, the final recall rate of easy items remained higher than that of difficult items. This outcome does not support the retrieval effort hypothesis, according to which more effort is required for a difficult task. As such, a better final correct recall rate should be produced. Therefore, Vaughn et al. (2013) argued that it is challenging to generate an effective mediator directly to connect the target words with the cue words. This suggests that for difficult items, we need additional encoding strategies (e.g., encoding items into meaningful sentences or using homophonic methods) to achieve the desired level of mastery.

While direct guidance may help learners to leverage the advantages of multiple retrieval practice entirely, the current study indicates that individual differences in ASE levels would affect learners’ persistence in repeated retrieval for tasks, thereby influencing final recall performance and confirming social cognitive theory’s point of view. Some researchers maintain that the performance of memory retrieval leads to cognitive pressure, which was called desirable difficulty; when learners find the RPG to be not direct and immediate or the task to be too hard, they may lose their motivation to learn (Parker and Roessger, 2020). In the present study, repeated retrieval made the retrieval tasks more difficult, especially, learners may realize that even they retrieved difficult items multiple times, it was still difficult to remember, but learners with different levels of ASE may have different perceptions. Learners with high self-efficacy are more willing to regard learning tasks or difficulties as challenges and to actively react to them; hence, they have higher engagement (Bates and Khasawneh, 2007). In contrast, learners with low ASE are prone to self-doubt or become bored when facing obstacles, which reduces the persistence of repeated retrieval. As for the motivation mechanism, horizontal and vertical studies have suggested that expectations affect academic achievement, while subjective value largely impacts choice, effort, and persistence (Nagengast et al., 2011; Guo et al., 2015). Learners with high ASE have greater task value and academic expectations for themselves, believing that they will have a positive impact on results; they are thus more driven to make learning choices and to perform better (Doménech-Betoret et al., 2017), which is reflected in tasks with different levels of difficulty. By contrast, in the present study, even if the participants were told that repeated retrieval could effectively promote memory, because of their low subjective evaluation of their own learning ability and low task value, learners with low ASE did not think they had a positive influence on the learning outcomes; they were less likely to practice repeated retrieval and, hence, their final recall performance was relatively poor. And we need to further consider what are the core beliefs behind these ideas? Wang et al. (2023) believed that when participants endorsed the belief that “effort and difficulty are indispensable in learning”, they were more confident in using retrieval practice effectively. And the more they believed that “effort, failure, and difficulties are signals of impossibility”, the less confident they became. The former believe that one can learn from failure and continue to persevere without hesitation, which is called growth mindset. Learners of growth mindset may have more intrinsic motivation to learn how to learn, better understand their memory, and adopt at least one effective intervention strategy (McCabe et al., 2021). This indicates that for learners with low ASE, the metacognitive level of instruction is not enough to encourage them to use cognitive strategies efficiently; it is necessary to boost their sense of efficacy in completing learning tasks to promote effective learning like growth-mindset training prior to teaching students about learning strategies and behavior change.

Overall, the findings demonstrate that RPG can improve learners’ SRL for tasks with different levels of difficulty, especially for learners with high ASE. To our knowledge, this research is the first study to explore the influencing factors of RPG in a laboratory experiment, which not only replicated the Karpicke’s research, also enriched the study of SRL of retrieval practice. The study helped us to understand learners’ retrieval practice decisions to some extent from a cognitive psychology perspective, increased the predictability of the actual use of retrieval practice and the impact of individuals with different ASE on RPG from an educational psychology perspective. The results provide valuable insights into factors that hinder or promote use of retrieval, and provide some support and suggestions for educational practice.

## 5. Limitations

The present study has some limitations, which also offer some prospects for future research. First of all, this study did not conduct a long-term recall test after a week, it is still unknown whether the recall performance after seven days remains the same as the performance after 15 min. A seven day interval recall may be better than a 15 min interval recall to test the retrieval effort hypothesis, according to which, the result of the cued-recall test would be an advantage for difficult tasks. Another issue is that we did not conduct a transfer session after a week without RPG, we do not know whether the effect of guidance could last, which future research can explore.

Secondly, there was no subjective survey to obtain participants’ views after completing the experiment, such as how effective they thought the RPG was and what made them persist or not persist in retrieving more than three times, what they mean when they report time and effort cost, what changes will happen to their attitudes when interventions occur in a laboratory or a classroom environment. Such a survey could have yielded a more intuitive understanding of students with different ASE, then we might be able to motivate them to optimize their use of retrieval practice.

Finally, we did not conduct experiments in the class context. Since retrieval practice strategies are often used by learners in self-testing before the exam, will students with low ASE be more active in retrieval practice in the context of exams? In addition, the materials used in this study differ from the learning materials in a class context. Interestingness may also affect the number of retrieval attempts by learners, thereby affecting recall performance. Those should be addressed in future research.

## 6. Conclusion

This study started from the intervention studies to enhance learners’ use of extraction exercises. Among numerous intervention studies, the results were inconsistent, therefore, it is necessary to consider influencing factors when providing interventions. The purpose of this research was to explore factors that may affect the effectiveness of RPG, including the difficulty of tasks and learners’ own ASE. The results indicated that RPG can effectively increase learners’ use of retrieval practice strategies and enhance learning effects. Under the conditions of providing sufficient time and immediate feedback, learners tend to perform more retrieval practice on a difficult task to maximize learning. At the same time, RPG is more effective for high ASE learners. Under the condition of providing guidance, learners with high ASE have higher persistence in repeated retrieval of tasks of different difficulty, and the learning effect is better.

The findings suggest that educators should provide students with timely and correct guidance on retrieval practice in regular education, to correct the misconception that retrieval practice is only a method of evaluating memory at the metacognitive level and “one-and-done” habit, also provide relatively sufficient time for difficult tasks to help them establish learning habits of repeated retrieval. At the same time, strengthen students’ self-efficacy, such as providing immediate feedback to let them know their progress, help them to experience a sense of achievement and control, and accurately monitor their mastery. It is also necessary to help students perceive and correct their automatic thinking and beliefs about difficulties, failures, and efforts, forming a growth mindset orientation. In other words, it is necessary to combine motivation, emotion, cognition, and metacognition for intervention, aiming to help students adopt efficient learning strategies (i.e., repeated retrieval practice) for more effective SRL.

## Data availability statement

The original contributions presented in the study are included in the article/supplementary material, further inquiries can be directed to the corresponding author.

## Ethics statement

The studies involving human participants were reviewed and approved by School of psychology, Fujian Normal University. The patients/participants provided their written informed consent to participate in this study.

## Author contributions

CL: Conceptualization, Data curation, Formal analysis, Investigation, Methodology, Software, Writing – original draft, Writing – review & editing. JZ: Funding acquisition, Project administration, Resources, Supervision, Validation, Visualization, Conceptualization, Methodology, Writing – review & editing.
